# A Rare Case of Medullary Carcinoma of the Ileum

**DOI:** 10.7759/cureus.3721

**Published:** 2018-12-11

**Authors:** Hector H Gonzalez, Simran Sidhu, Todd Eisner

**Affiliations:** 1 Internal Medicine, Florida Atlantic University Charles E. Schmidt College of Medicine, Boca Raton, USA; 2 Gastroenterology, Boca Raton Regional Hospital, Boca Raton, USA

**Keywords:** medullary carcinoma, tumor, small intestine

## Abstract

Medullary carcinoma of the small intestine is an exceedingly rare tumor. These tumors account for less than 0.04% of all colorectal cancers and only one case to date has been reported in the ileum. Although the clinical manifestations can be consistent with signs of intestinal obstruction, often times they are discovered incidentally in an asymptomatic patient. Major contributing risk factors to the development include long standing inflammation such as Crohn's disease, and other chronic inflammatory illnesses. Tumor markers and imaging can aid in the diagnosis, however biopsy is needed for definitive diagnosis. Despite the fact that the development of these tumors in the ileum is rare, further enhancement of awareness can aid in the appropriate early detection and appropriate treatment modalities.

## Introduction

Medullary carcinoma of the small intestine is a unique histopathological subtype of colorectal cancers whose underlying mechanism is unknown. The World Health Organization (WHO) recognized this rare condition as a separate entity in colorectal cancers in hopes to better specify treatment [1]. Medullary carcinoma of the colon is a rare neoplasm accounting for less than 0.04% of all colorectal cancers and only one case has been reported in the ileum, making the following case report exceptionally rare.

## Case presentation

**Figure 1:**
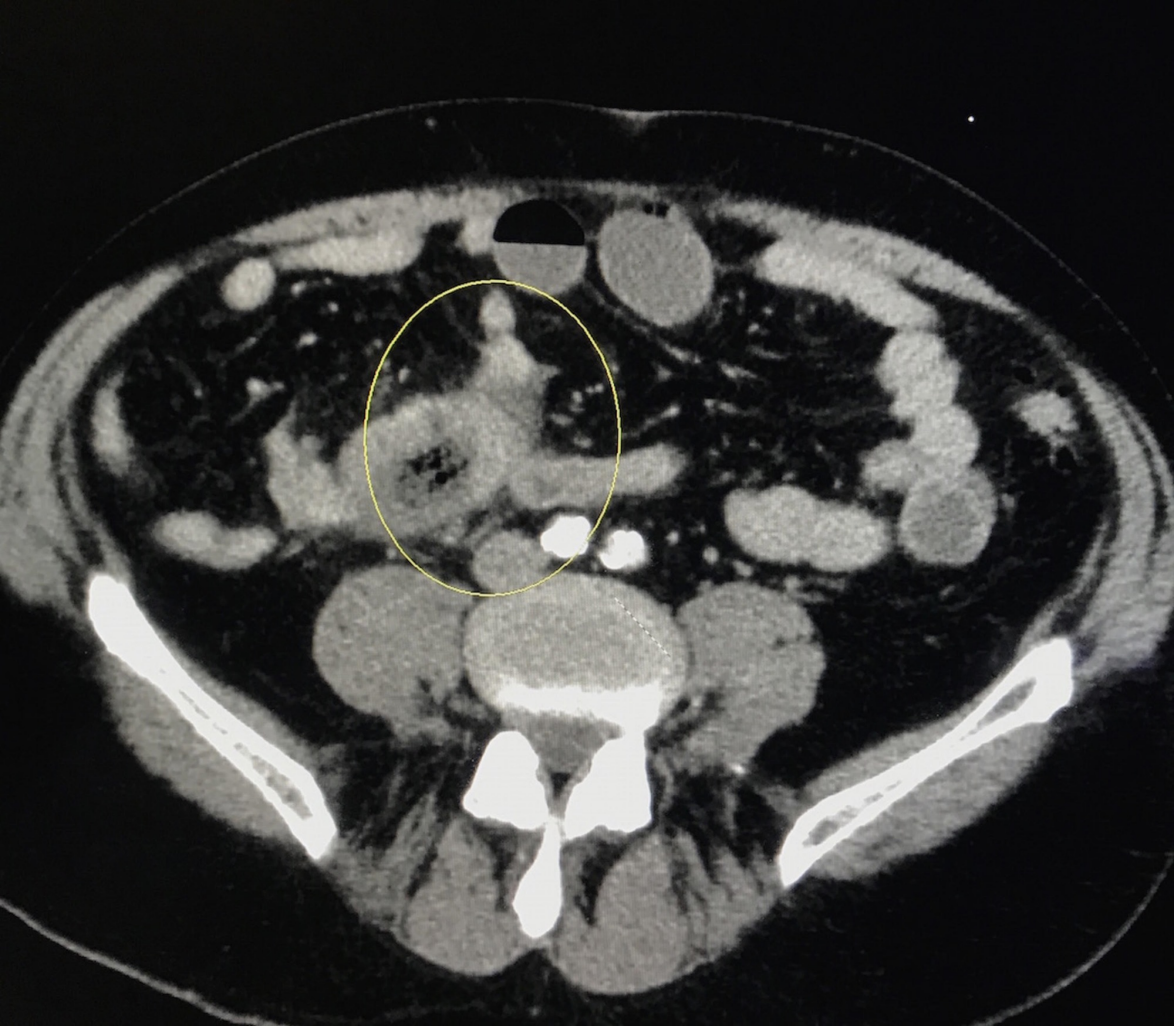
Computed tomography of the abdomen and pelvis demonstrating intermediate grade small bowel obstruction.

**Figure 2:**
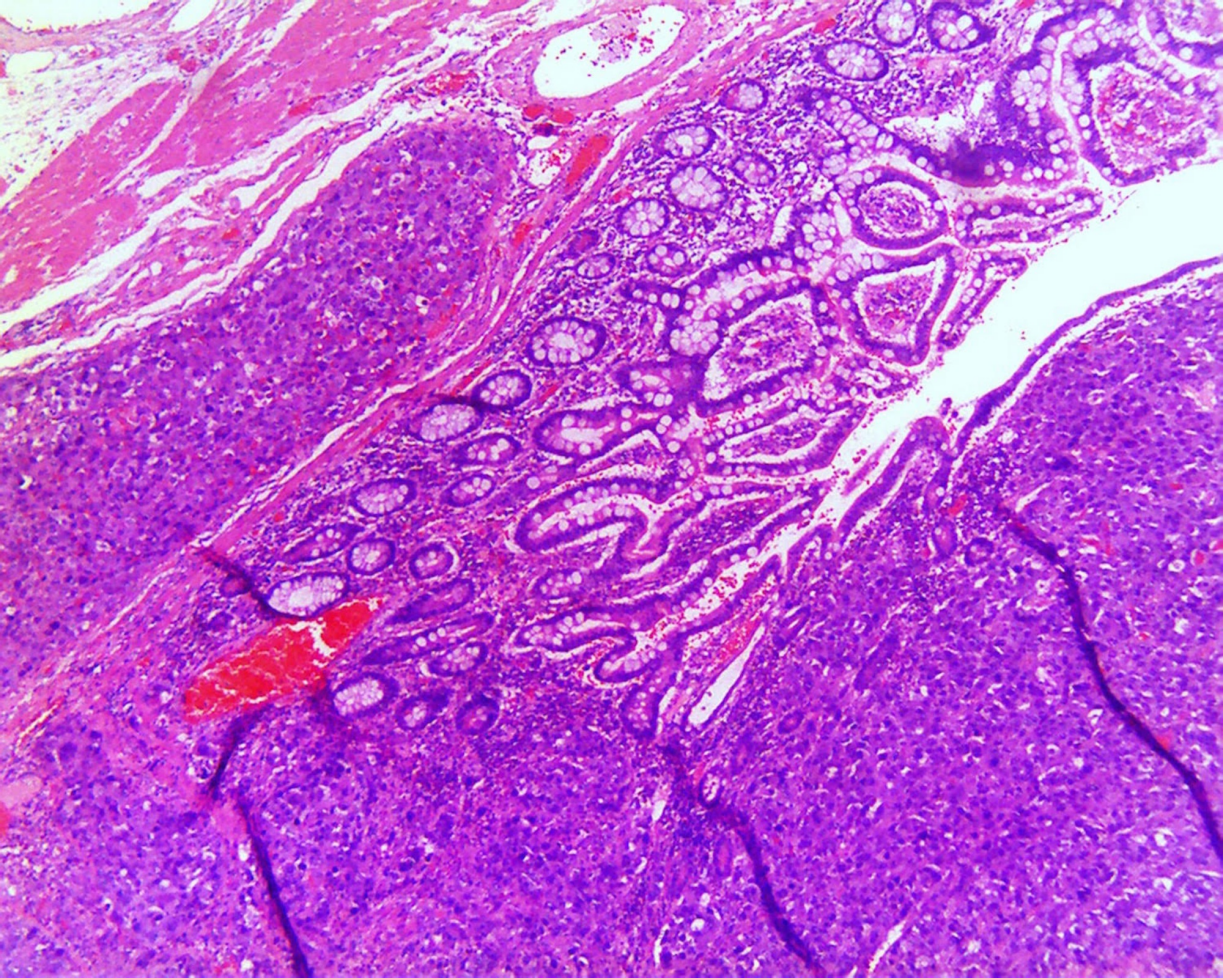
Histologic image showing sheets of syncytial cells with abundant cytoplasm and prominent nucleoli.

**Figure 3:**
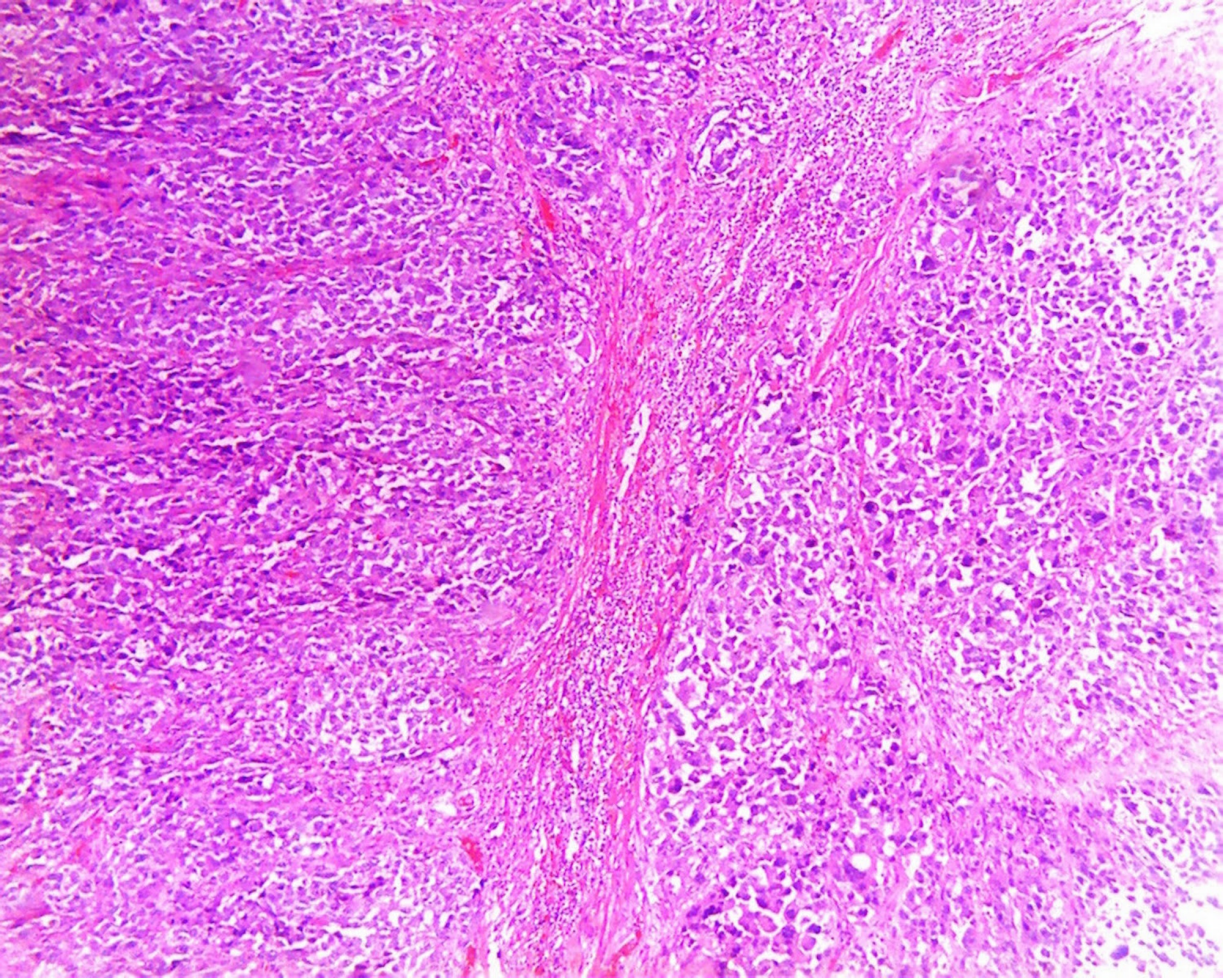
Hematoxylin and eosin stain demonstrating tumor invading into muscularis propia.

A 70-year-old male with past medical history of psoriasis, diabetes mellitus, and hypertension, presented to the emergency department with diffuse, sharp, abdominal pain of four days duration. Physical exam was remarkable only for diffuse tenderness to palpation of abdomen, and mild abdominal distention. Laboratory data showed white blood cells (WBC) of 13.4 K/MCL (Normal 4–10 K/MCL), lipase 18 U/L (Normal 0–160 U/L). Colonoscopy performed two years ago was unremarkable. Computed tomography (CT) abdomen/pelvis showed intermediate grade small bowel obstruction, without evidence of any mass (Figure 1). Exploratory laparoscopy with ileal mass resection was performed. Upon gross examination of resected segment, a necrotic mass measuring 4.8 cm x 3.9 cm x 3.8 cm was visualized. Specimen pathology showed high-grade medullary carcinoma of the ileum with angiolymphatic invasion (Figure 2). The carcinoma invaded through the muscularis propia into the periileal adipose tissue (Figure 3). The tumor stage was pT3N0M0. Immunohistochemistry was positive for epithelial membrane antigen (EMA), and pancytokeratin. Ki-67 index was 80%. CDX-2, CD56 synaptophysin, and chromogranin were negative. The patient was treated with local excision and radiation therapy and instructed on appropriate follow-up. On follow-up, the patient was noted to be free of disease without any adjuvant therapy one year later.

## Discussion

Medullary carcinoma is a rare colorectal neoplasm which typically manifests in the colon, occurring with more frequency in the ascending colon. This neoplasm occurs more frequently in women, and arises in the seventh decade of life [2]. Studies have suggested an association between microsatellite instability and the predisposition to developing this neoplasm [3-5]. Main histopathology consists of intra-tumor lymphocytic infiltration and poor gland differentiation. The exact underlying significance of this characteristic histological appearance is poorly understood, however these infiltrating lymphocytes have shown to have a low proliferative capacity. Cancers that exhibit DNA replication errors have been linked to being targets for cytotoxic T cells, which stems from the expression of neoantigens that have been translated from mutated genes [4]. However, the susceptibility to cytotoxic T cells has been circumvented through the loss of HLA genes by these cancers rendering them resistant [4]. Current literature has correlated lack of keratin-20 in medullary neoplasms with variable expression of CDX-2 [2]. Despite the propensity for medullary carcinomas to exhibit a spectrum of genetic variability, the overall prognosis has been shown to be more favorable than other colorectal carcinomas.

Major factors contributing to the development of medullary carcinoma include those associated with chronic underlying inflammation which was not noted in our patient. The presence of inflammatory conditions such as ulcerative colitis, celiac or Crohn's disease may serve as triggers for the development of this neoplasm [6]. The predisposition for development of medullary carcinoma in celiac disease increases with both age and delayed diagnosis due to lack of treatment [2]. Our patient presented with features of intestinal obstruction with laparoscopy revealing an ileal mass. It is important to maintain a differential for carcinoma when confronted with colonic masses such as suspected intra-abdominal abscesses, and inflammation as these can mimic more sinister underlying gastrointestinal conditions (Abstract of poster presentation: Odufalu FD, Goldkamp W. When a Lap Band Goes Rogue. Oct 8, 2018).

Treatment modalities aimed at eradication medullary carcinoma are yet to be established. Medullary carcinoma typically presents with evidence of local advanced disease, with outcomes showing high mortality 30 days after resection [7]. Current literature has shown favorable outcomes with resection in those without metastasis, with mismatch repair deficiency serving as a favorable prognostic marker [7]. The treatment with neoadjuvant chemotherapy has yet to be established, and limited evidence exists to suggest any mortality benefit. Studies have noted BRAFV600E mutation to not only manifest more frequently in those with medullary carcinoma but possibly serving to predict response to chemotherapy as a treatment modality [7]. This mutation confers a diminished response to chemotherapy in advanced case of colon cancer and implies a poor prognosis.

## Conclusions

This report presents the second case of medullary carcinoma of the ileum in the English literature. Despite the rarity of this subclass of colorectal cancer, one must keep this in the differential especially in a patient with history of chronic inflammatory intestinal disease. It is clear that additional diagnostic and treatment studies need to be conducted, but until then, the treatment for medullary carcinoma of the ileum will remain inconsistent due to a lack of standardized diagnostic criteria.
